# Prediction of functional consequences of the five newly discovered *G6PD* variations in Taiwan

**DOI:** 10.1016/j.dib.2019.104129

**Published:** 2019-06-11

**Authors:** Yen-Hui Chiu, Yu-Ning Liu, Hsiao-Jan Chen, Ying-Chen Chang, Shu-Min Kao, Mei-Ying Liu, Ying-Yen Weng, Kwang-Jen Hsiao, Tze-Tze Liu

**Affiliations:** a Department of Education and Research, Taipei City Hospital, Taipei, Taiwan; b Department of Biotechnology and Pharmaceutical Technology, Yuanpei University of Medical Technology, Hsinchu, Taiwan; c Neonatal Screening Center, The Chinese Foundation of Health, Taipei, Taiwan; d Cancer Progression Research Center, National Yang-Ming University, Taipei, Taiwan; e Preventive Medicine Foundation, Taipei, Taiwan

**Keywords:** G6PD deficiency, Mutation analysis, *In silico* analysis, Structural predication

## Abstract

Glucose-6-phosphate dehydrogenase deficiency (G6PD deficiency; OMIM #300908) is the most common inborn error disorders worldwide. While the G6PD is the key enzyme of removing oxidative stress in erythrocytes, the early diagnosis is utmost vital to prevent chronic and drug-, food- or infection-induced hemolytic anemia. The characterization of the mutations is also important for the subsequent genetic counseling, especially for female carrier with ambiguous enzyme activities and males with mild mutations. While multiplex SNaPshot assay and Sanger sequencing were performed on 500 G6PD deficient males, five newly discovered variations, namely c.187G > A (p.E63K), c.585G > C (p.Q195H), c.586A > T (p.I196F), c.743G > A (p.G248D), and c.1330G > A (p.V444I) were detected in the other six patients. These variants were previously named as the Pingtung, Tainan, Changhua, Chiayi, and Tainan-2 variants, respectively. The *in silico* analysis, as well as the prediction of the structure of the resultant mutant G6PD protein indicated that these five newly discovered variants might be disease causing mutations.

**Table:** Specifications table

Subject area	Genetics, Genomics and Molecular Biology
More specific subject area	Inborn errors of metabolism
Type of data	Tables, Figures
How data was acquired	DNA sequencing using 3730xl Genetic Analyzer (Thermo Fisher Scientific, Waltham, MA, USA), mutation severity prediction softwares, structural effect prediction software
Data format	Analyzed
Experimental factors	DNA extracted from dried blood spot used in newborn screening
Experimental features	Bioinformatic tools
Data source location	Taiwan
Data accessibility	Provided within this article
Related research article	Chiu YH, Chen HJ, Chang YC, Liu YN, Kao SM, Liu MY, Weng YY, Hsiao KJ, Liu TT. Applying a multiplexed primer extension method on dried blood spots increased the detection of carriers at risk of glucose-6-phosphate dehydrogenase deficiency in newborn screening program. Clin. Chim. Acta 495 (2019) 271–277. https://doi.org/10.1016/j.cca.2019.04.074[1].

**Table:** 

**Value of the Data** • This study extends the *G6PD* mutation spectrum. • The three-dimensional structure illustrates the importance of the amino acid residues related to the function of the G6PD protein. • The *in silico* analysis served as a tool in determining the functional consequence of the mutations, making it potentially valuable for primary care as well as research processes.

## 1 Data

**Fig. 1:**
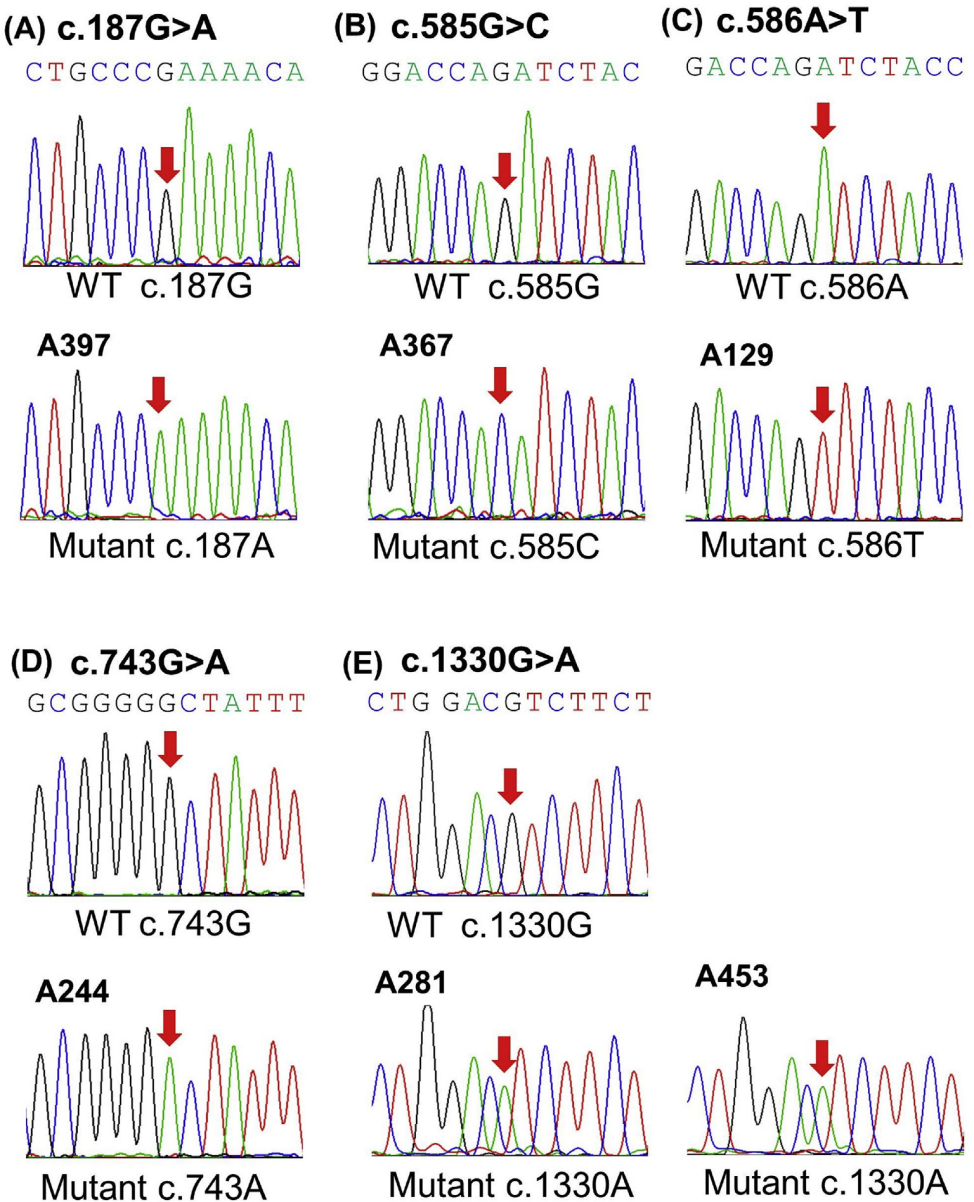
Detection of five new *G6PD* variations by Sanger sequencing. *G6PD* gene sequence showed the wild type sequence with variants of different individuals. (A) c.187G > A in patient A397, (B) c.585G > C in patient A367, (C) c.586A > T in patient A 129, (D) c.743G > A in patient A244 and (E) c.1330G > A in patients A281 and A453. The red arrows showed substitution in a hemizygous state in the missense mutations observed.

This dataset presented the *in silico* and structural analysis of the five newly discovered variations, namely c.187G > A (p.E63K), c.585G > C (p.Q195H), c.586A > T (p.I196F), c.743G > A (p.G248D), and c.1330G > A (p.V444I) (Fig. 1), detected in the six Taiwanese G6PD deficient patients using Sanger Sequencing (Table 1).

**Table 1:** G6PD activity in newborn screening and following referral for patients carrying newly discovered *G6PD* variations.

Patient Number	A129	A244	A281	A367	A397	A453
Sex	Male	Male	Male	Male	Male	Male
Place of Birth	Changhua	Chiayi	Tainan	Tainan	Pingtung	Tainan
Age at newborn screening (day)	2	2	2	2	3	3
G6PD activity in newborn screening (U/gHb)^a^	0.2	5.5	5.3	1.7	5.7	5.1
Age when confirmed (day)	34	9	22	15	14	11
Confirmed G6PD activity (U/gHb)^b^	0.1	6.1	5.5	0.2	8.6	6.5
Variation found	c.586A > T	c.743G > A	c.1330G > A	c.585G > C	c.187G > A	c.1330G > A

a Clinical referral was recommended for those enzyme activity ≦6.0 U/gHb.

b The confirmed diagnosis was performed through a quantitative enzyme activity assay by using fresh whole blood. G6PD-deficiency would be suggested for those with G6PD activity ≦10.0 U/gHb.

**Fig. 2:**
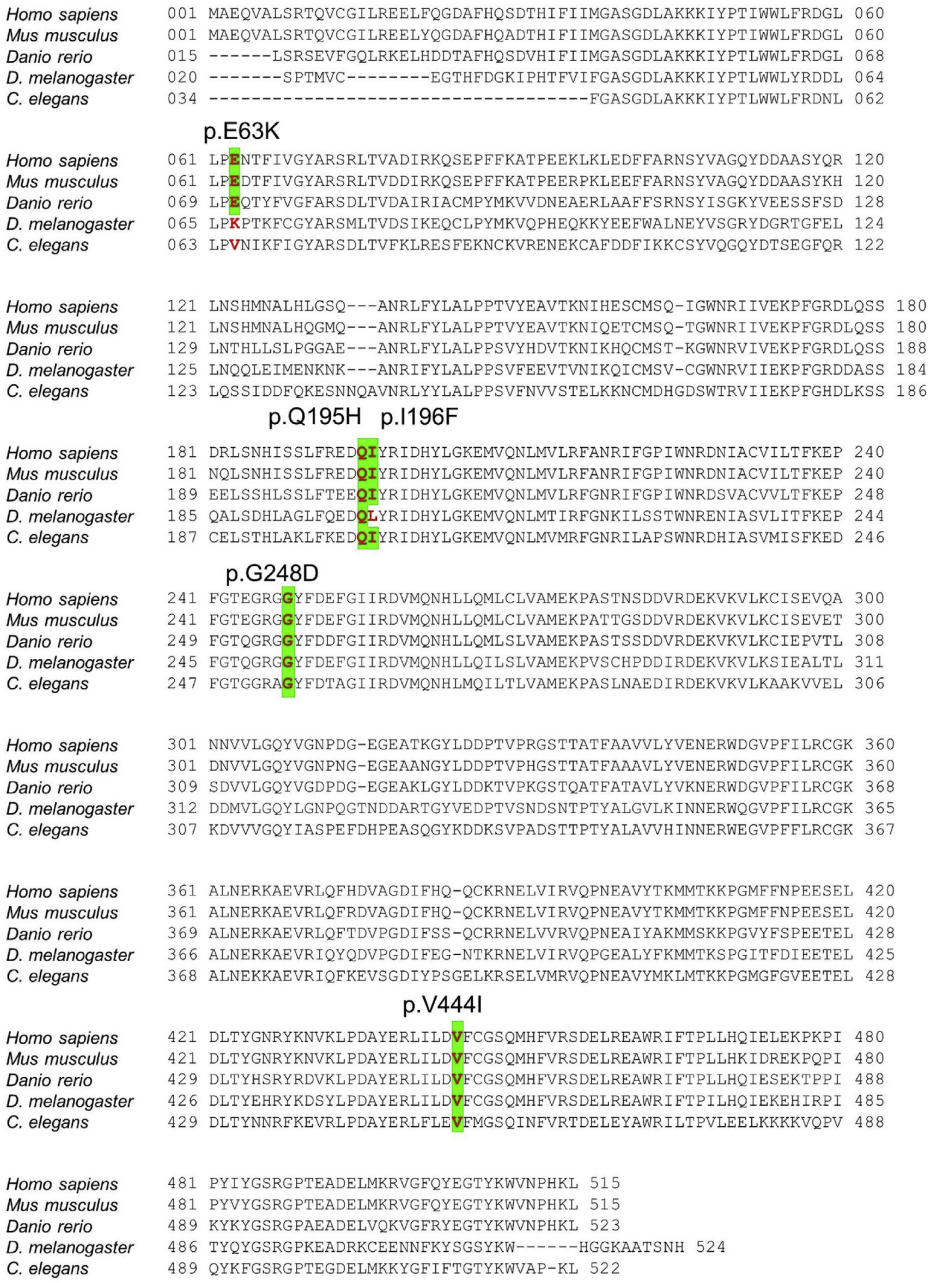
The similarity alignment of G6PD proteins across different species. The red characters show the corresponding positions of the five substitutions between species whereas the conserved residues were outlined in green box. The species abbreviations are: *D. melanogaster*, *Drosophila melanogaster*; *C. elegans*, *Caenorhabditis elegans.*

**Fig. 3:**
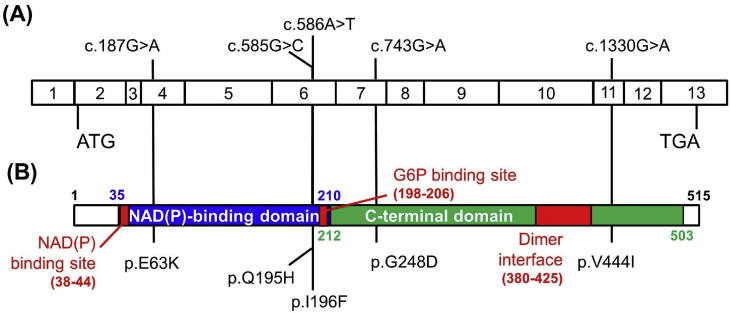
Schematic representation of alterations in G6PD coding regions and protein functional domains. (A) The coding region of the *G6PD* gene containing 13 exons. (B) The G6PD protein of 515 amino acids contains two binding domains, namely NAD(P)-binding domain (blue box, amino acids 25–210) and *C*-terminal domain (green box, amino acids 212–503), and two binding sites, namely NAD(P) binding site (left red box, amino acids 38–44) and G6P-binding site (middle red box, amino acids 198–206), and one dimer interface (right red box, amino acids 380–425). The five mutations were highlighted in black in the coding region and protein domains.

**Fig. 4:**
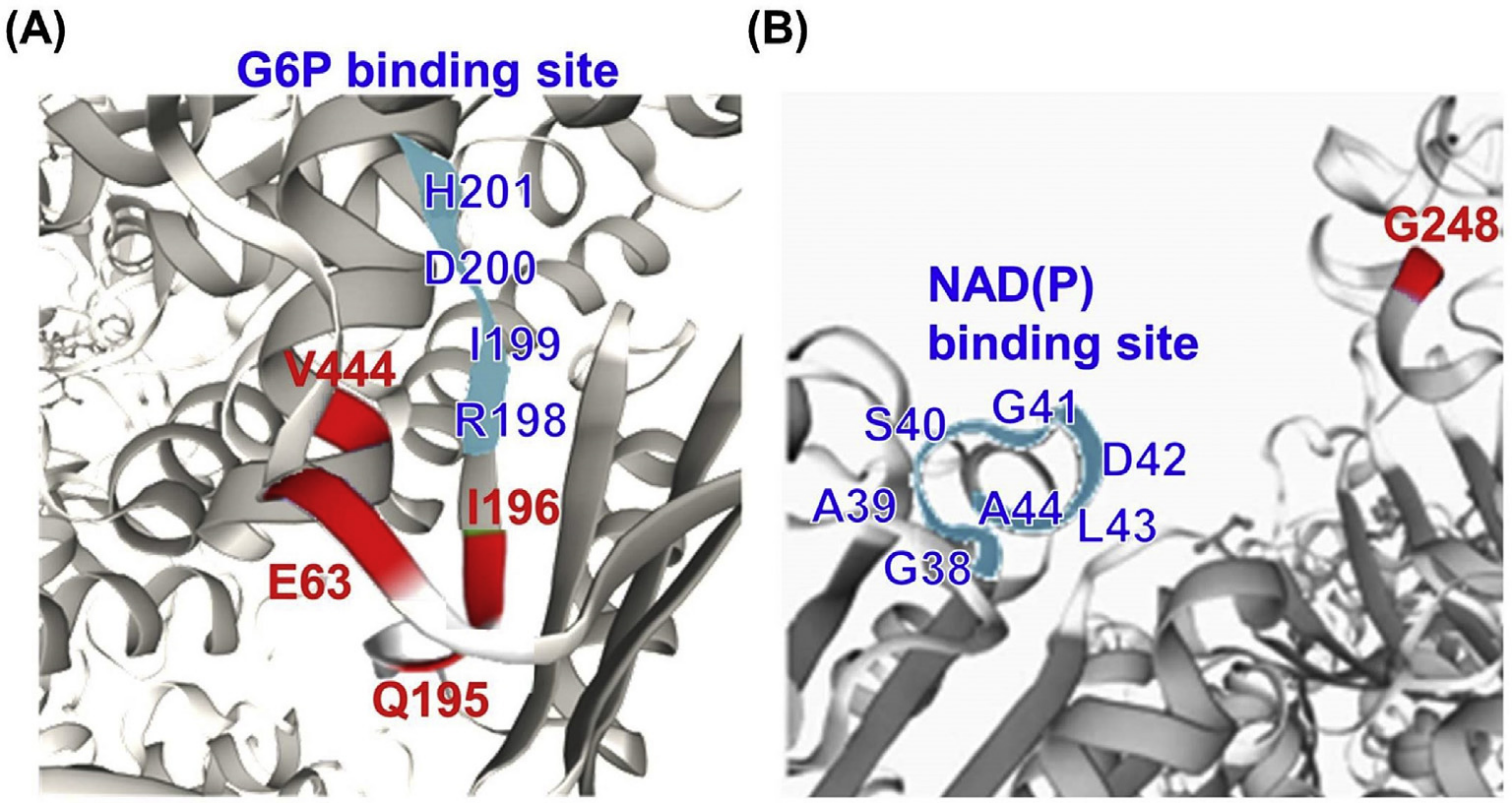
Close-up views of the ribbon diagram of human G6PD as generated by Swiss PDB viewer. (A) The 3D model structure of G6PD closed to the G6P-binding site, and the Glu63, Gln195, Ile196 and Val444 residuals. (B) A close-up view of G6PD protein contains the NAD(P)-binding site and Gly248 residual. The G6P- and NAD(P)-binding sites were highlighted in cyan, while the residuals were presented in red.

The comparison sequence of these variants in G6PD protein of different species [2], including *Homo sapiens*, *Mus musculus*, *Danio rerio* (zebrafish), *Drosophila melanogaster* (fruit fly), and *Caenorhabditis elegans* were presented in Fig. 2. The *in silico* analysis using SIFT [3], PolyPhen-2 [3], Mutation Taster [4] and Slicing Finder [5] softwares, as well as the conservation between species and allele frequency in Taiwanese population [6] were summarized in Table 2. Furthermore, the amino acid alterations were presented in the functional domains [7] (Fig. 3) and in partial 3D model of G6PD [8] (Fig. 4). The structure of the resultant mutant G6PD protein were analyzed by HOPE, Have yOur Protein Explained [9] (Table 3).

**Table 2:** The severity prediction for five newly discovered *G6PD* missense variations.

Nucleotide substitution	Amino acid substitution	SIFT	PolyPhen-2	Mutation Taster	Splicing finder	Conservation^a^	Allele Frequency^b^	Predicted Class^c^
c.187G > A	p.E63K	Tolerated	Benign	Disease causing	Potential alteration	Moderately	<2/1417^d^	III-IV
c.585G > C	p.Q195H	Damaging	Probably damaging	Disease causing	Potential alteration	Highly	<1/1000	II
c.586A > T	p.I196F	Damaging	Probably damaging	Disease causing	Potential alteration	Highly	<1/1000	II
c.743G > A	p.G248D	Damaging	Probably damaging	Disease causing	Probably no impact	Highly	<1/1000	III
c.1330G > A	p.V444I	Tolerated	Possibly damaging	Disease causing	Potential alteration	Highly	<1/1000	III

a Sequence comparison between *Homo sapiens*, *Mus musculus*, *Danio rerio* (zebrafish), *Drosophila melanogaster* (fruit fly), and *Caenorhabditis elegans* and *Saccharomyces cerevisiae* as shown in Fig. 2.

b Allele frequency in Taiwanese population (https://taiwanview.twbiobank.org.tw/browse38, accessed on 25 April 2019) [6].

c Classification of *G6PD* variants in the study according to the WHO definition [7].

d Two alleles in 1417 people with indeterminate sex.

**Table 3:** Structure prediction of the *G6PD* variations by HOPE algorithm.

Mutants	Structure prediction by HOPE algorithm^a^
p.E63K	The wide-type residue forms a salt bridge with arginine at position 104. The difference in charge will disturb the ionic interaction made by the original, wild-type residue.
p.Q195H	The wild-type residue forms a hydrogen bond with arginine at position 192. The size difference between wild-type and mutant residue makes that the new residue is not in the correct position to make the same hydrogen bond as the original wild-type residue did.
p.I196F	The mutant residue is bigger than the wild-type residue and is located in a domain that is important for the activity of the protein and in contact with residues in another domain. The mutation can affect this interaction and as such affect protein function.
p.G248D	The wild-type residue is a glycine, the most flexible of all residues. This flexibility might be necessary for the protein's function. Mutation of this glycine can abolish this function.
p.V444I	The mutant residue is bigger than the wild-type residue and is located in a domain that is important for binding of other molecules. The mutation might affect this interaction and thereby disturb signal transfer from binding domain to the activity domain.

a Using software Have yOur Protein Explained (HOPE, http://www.cmbi.ru.nl/hope/) [9].

## 2 Experimental design, materials and methods

### 2.1 Mutation identification: sanger sequencing

In 500 G6PD-deficient male newborns detected by G6PD enzyme activity assay [10], nine of which do not carry any of the 21 common mutations described in Taiwan and Southeast Asia using multiplex SNaPshot assay [1]. Their dried blood spots used in newborn screening were subsequently subjected to mutational analysis by sequencing. The whole coding exons and exon-intron boundary sequences of *G6PD* gene were amplified and analyzed by forward and reverse Sanger sequencing. Putative mutations were confirmed by sequencing of an independent PCR product. The study protocol was reviewed and approved by the Institutional Review Board of Taipei City Hospital, Taiwan.

### 2.2 Sequence alignments between species

Conservation of the peptide sequence around the affected residues was assessed by alignment of orthologous and human G6PD sequences with ClustalW2, [2].

### 2.3 Severity prediction and allele frequency in population

Different online algorithms were used to predict the functional consequences of the five variants. The *in silico* analyses were performed using the SIFT [3], PolyPhen-2 [3], MutationTaster2 [4], and Human Splicing Finder [5] programs. Furthermore, the allele frequency of the alterations in Taiwanese population was listed as provided in Taiwan Biobank [6].

### 2.4 Distribution of mutations along the coding region and protein sequence

Distribution of alterations was highlighted in the coding region and the functional domains [7]. The A at the ATG translational initiation codon was numbered as 1 in reference accession number NM_001042351. The amino acid numbers were counted from the *N*-terminal Met of human G6PD protein.

### 2.5 3D structure model of wide type G6PD protein

The 3D structure of *G6PD* variations observed in this study were presented based on the X-ray crystal structure available at the Protein Data Bank from human G6PD protein (PDB code 1QKI) [8].

### 2.6 Prediction of structural effects of variations

When protein structure is important to predict the effects of variants [11], effect of mutations over G6PD protein structure was determined using HOPE (Have yOur Protein Explained) software [9].
